# First-line latanoprost therapy in ocular hypertension or open-angle glaucoma patients: a 3-month efficacy analysis stratified by initial intraocular pressure

**DOI:** 10.1186/1471-2415-10-4

**Published:** 2010-02-24

**Authors:** Philippe Denis, Christophe Baudouin, Alain Bron, Jean-Philippe Nordmann, Jean Paul Renard, Jean François Rouland, Eric Sellem, Mourad Amrane

**Affiliations:** 1 Ophthalmology Department, Hôpital Edouard Herriot, 5, Place d'Arsonval, 690437 Lyon Cedex 03, France; 2 Ophthalmology Department 3, Centre Hospitalier National des Quinze-Vingts, 28, Rue de Charenton, Paris Cedex 12, France; 3 Ophthalmology Department, Hôpital Général, 3, Rue du Faubourg Raines, 21033 Dijon, France; 4 Ophthalmology Department 2, Centre Hospitalier National des Quinze-Vingts, 28, Rue de Charenton, Paris Cedex 12, France; 5 Ophthalmology Department, Hôpital d'Instruction des Armées du Val de Grâce, 74, Boulevard du Port Royal, Paris Cedex 05, France; 6 Ophthalmology Department, Hôpital Huriez, 1, Place de Verdun, 59037 Lille, France; 7 Centre Ophtalmologique Kléber, 50, Cours Franklin Roosevelt, 69006 Lyon, France; 8 1 allée Giuseppe Verdi, 94350 Villiers Sur Marne, France

## Abstract

**Background:**

Prospective, multicenter, randomized, double-masked trials have shown latanoprost instilled once daily to be at least as effective as and generally superior to timolol administered twice daily and to be as effective as other frequently prescribed prostaglandin analogues. This study prospectively assessed the efficacy of latanoprost monotherapy in a large cohort of treatment-naive patients with a broad range of baseline intraocular pressure (IOP) levels treated in actual clinical practice settings.

**Methods:**

This prospective, open-label, multicenter, uncontrolled, phase IV study included treatment-naive ocular hypertension or open-angle glaucoma subjects initiating latanoprost once daily (evening). IOP levels were measured at baseline and after 1 and 3 months. The primary efficacy outcome was mean change in IOP from baseline to month 3. Analyses were stratified by baseline IOP: ≥ 20 and <24 mmHg *vs *≥ 24 mmHg.

**Results:**

Efficacy analyses (intent to treat) included 572 subjects: 20 to <24 mmHg group, N = 252; ≥ 24 mmHg group, N = 320. Mean baseline IOP levels were 22.2 ± 0.9 mmHg and 26.7 ± 2.8 mmHg, respectively. At month 3, significant IOP reductions were seen in both groups (p < 0.0001, within-group differences); reductions were smaller in the 20 to <24 mmHg group (-6.3 ± 2.4 *vs *-9.2 ± 3.7 mmHg, respectively; -28.0 ± 10.6% *vs *-34.1 ± 11.9%, respectively). An IOP reduction of ≥ 30% from baseline to month 3 was noted in 48.4% and 65.6% of subjects, respectively (p < 0.0001). At month 3, targets IOPs of ≤ 18 mmHg were achieved by ≥ 70% of subjects in both groups. Latanoprost was well tolerated with an adverse event profile similar to that reported in the literature.

**Conclusions:**

This "real world" study found once-daily latanoprost to be effective and safe in treatment-naive ocular hypertension or open-angle glaucoma patients. Patients with baseline IOP levels of 20 to <24 mmHg as well as ≥ 24 mmHg benefitted from initial latanoprost therapy.

**Trial Registration:**

Trial Registration Number: NCT00647101

## Background

Glaucoma is among the leading causes of blindness worldwide [1], and elevated intraocular pressure (IOP) is a major risk factor for progression of both ocular hypertension and glaucoma [2]. Reducing IOP prevents or delays the onset of open-angle glaucoma in patients with ocular hypertension [3] and slows progression among those with open-angle glaucoma [4-8].

Treatment to reduce IOP levels commonly begins with topical ocular hypotensive agents. Among these, latanoprost, which became available in 1996 and was approved by the European Agency for the Evaluation of Medicinal Products as first-line treatment in March 2002, is one of the most frequently prescribed and has been shown to be at least as effective as or superior to the beta-blocker timolol [9-13]. A pooled analysis [13] of data from eight prospective, randomized, parallel-group trials (five of which were double-masked) found latanoprost reduced diurnal IOP levels statistically significantly more than timolol in a global population of ocular hypertension and open-angle glaucoma patients (N = 1389). Sub-group analyses demonstrated that latanoprost was effective across racial and ethnic groups and resulted in similar mean diurnal IOP reductions in those with and without prior ocular hypotensive treatment other than prostaglandins.

While findings of individual prospective, randomized, parallel-group, double-masked clinical trials - the gold standard for evaluating new therapies - and pooled analyses across trials provide convincing evidence of the efficacy and safety of latanoprost, their designs are restrictive and do not reflect conditions found in routine clinical practice settings. In particular, these trials generally recruited both treatment-naive and previously treated patients; set strict inclusion and exclusion criteria, often stipulating that baseline IOP levels be ≥ 22 mmHg; established a washout period for patients under treatment at screening; and required frequent patient monitoring. Thus, it remains of interest to prospectively assess the efficacy of latanoprost monotherapy in a large cohort of treatment-naive ocular hypertension and open-angle glaucoma patients with a broader range of baseline IOP levels treated in actual clinical practice settings.

The primary purpose of the present study was to conduct such an assessment in patients treated in ophthalmology practices in France who were followed for 3 months after being prescribed first-line latanoprost 0.005% administered once daily. In order to determine whether response to latanoprost differs by presenting IOP level, it was predetermined that analyses would be stratified by baseline IOP dichotomized as ≥ 20 and <24 mmHg (20 to <24 mmHg group) *vs *≥ 24 mmHg (≥ 24 mmHg group). The cut point of 24 mmHg was considered to be clinically relevant and to distinguish roughly between those with very high *vs *moderately high IOP levels. The Collaborative Normal-Tension Glaucoma Study [14] included only patients with no recorded IOP over 24 mmHg in either eye at screening.

## Methods

### Study design

This was a prospective, 3-month, open-label, multicenter, uncontrolled, phase IV study (NCT00647101) conducted in 258 ophthalmology practices in France. The final protocol and informed consent documents were reviewed and approved by the Independent Ethics Committee (*Comité Consultatif de Protection des Personnes se prêtant à une Recherche Biomédicale de Lyon, France*). The study complied with local French laws, with the International Conference on Harmonization Guidelines, and with the Declaration of Helsinki Guidelines. Written informed consent was obtained from each subject prior to study enrollment. We certify that all applicable institutional and governmental regulations concerning the ethical use of human volunteers were followed during this research.

### Subjects

Eligible subjects were at least 18 years of age and had a baseline (initial visit) IOP of ≥ 20 mmHg related to a diagnosis of unilateral or bilateral ocular hypertension or open-angle glaucoma (primary open-angle glaucoma, pseudoexfoliation glaucoma, or pigmentary glaucoma) following a visual field examination. Only subjects who, in the investigator's opinion, required initiation of ocular hypotensive treatment and who had never been treated for ocular hypertension or open-angle glaucoma were eligible. In an effort to balance enrollment across IOP strata, investigators were instructed to enroll one subject in each stratum before enrolling additional subjects in either stratum.

Subjects were excluded if they had traumatic, inflammatory, or neovascular glaucoma; had any ophthalmic or systemic disorder, including uncontrolled asthma, that, in the opinion of the investigator, would prevent study entry; had a known hypersensitivity to benzalkonium chloride or any other component of latanoprost; or had participated in another clinical trial within 30 days prior to the enrollment visit. In addition, women of childbearing potential who were not using adequate contraceptive methods or who were pregnant or nursing were not included.

### Treatment and assessments

Subjects were assessed for eligibility at the baseline visit. Demographic information, ocular and medical histories, and concomitant medications were documented; best-corrected visual acuity was measured; biomicroscopy, ophthalmoscopy, an eyelid examination, and fundoscopy were performed; and stage of open-angle glaucoma was classified as early, moderate, or severe. A visual field examination was conducted at baseline or within the following 1 month unless such an examination had been conducted during the 6 months prior to baseline. IOP was measured prior to pupil dilation using an air-pulsed tonometer (three measurements; mean IOP value used in analyses) or a calibrated Goldmann applanation tonometer (single measurement).

All subjects were prescribed latanoprost 0.005% to be instilled once daily in the evening. Other ocular hypotensive medications were prohibited during the study.

Follow-up visits were scheduled after 1 and 3 months of latanoprost treatment. At each visit, concomitant medications were recorded, and biomicroscopy, ophthalmoscopy, and an eyelid examination were performed. IOP was measured at the same time (± 1 hour) as at the baseline visit and prior to pupil dilation. The same calibrated device (air-pulsed tonometer or Goldmann applanation tonometer) was used for the same subject at all visits. As at the baseline visit, three measurements were made for subjects evaluated with an air-pulsed tonometer (mean IOP value used in analyses) and a single measurement was made for those assessed with a Goldmann applanation tonometer. At month 3, best-corrected visual acuity was measured and fundoscopy was performed. At the 1 and 3 month visits, subjects were asked a single question about how often they forgot to take their latanoprost, and responses were classified as "took every day," "rarely forgot," and "forgot sometimes."

All adverse events, whether observed or volunteered, were recorded at each follow-up visit. The severity of events (mild, moderate, or severe) and the investigator's opinion about whether the event was related to study drug were recorded. Serious adverse events were defined as those that were life-threatening, required inpatient hospitalisation/prolongation of hospitalisation, caused persistent or significant disability/incapacity, or resulted in congenital anomaly/birth defect or death. Adverse events were followed until they resolved or stabilised. As part of adverse event reporting, investigators were instructed to question patients concerning whether they had used any bottle of latanoprost beyond its expiration date, i.e., for >28 days after opening. Any such extended use was reported as an adverse event, and the investigator also documented whether any related clinical problem had occurred.

### Endpoints and analyses

Efficacy analyses used only IOP measurements of one study eye per subject. If both eyes of a subject were eligible, the eye with the highest IOP at baseline was considered the study eye; if the baseline IOP was the same in both eyes, the right eye was considered the study eye.

The primary efficacy endpoint was mean change in IOP from baseline to month 3. Secondary efficacy endpoints included mean change in IOP from baseline to month 1; mean percent change in IOP from baseline to months 1 and 3; percentage of subjects achieving ≥ 10% and ≥ 30% reductions in IOP from baseline to month 3; and percentage of subjects achieving target IOP levels of ≤ 21, ≤ 18, and ≤ 15 mmHg at months 1 and 3.

Efficacy analyses were stratified by baseline IOP level (20 to <24 *vs *≥ 24 mmHg) and were conducted in the intent-to-treat population which included all subjects who met entry criteria, who instilled ≥ 1 dose of study medication, and for whom a baseline IOP measurement and at least 1 on-treatment IOP measurement was recorded. In efficacy analyses, the last observation carried forward method was used to impute missing data. Safety analyses included all subjects who instilled ≥ 1 dose of study medication.

The statistical significance of within-stratum changes in mean IOP levels from baseline to months 1 and 3 was evaluated using paired *t *tests. The significance of between-strata differences in frequencies of subjects achieving prespecified percentage IOP reductions and target IOP levels was assessed using chi-square tests. The two-sided significance level was set at the 0.05 level.

Multiple correspondence analyses were performed to identify factors predicting response to treatment defined as a ≥ 30% reduction in IOP from baseline to month 3. Potential predictors included IOP stratum (20 to <24 *vs *≥ 24 mmHg); age (<55, 55 to 65, >65 years); gender; ocular hypertension *vs *open-angle glaucoma; myopia (>-6 diopters, ≤-6 diopters, no myopia) presence/absence of diabetes, sleep disorders, use of antihypertensive medication, obesity, and family history of ocular hypertension or glaucoma; tobacco use within prior year (yes/no); and compliance (good/poor). Forward stepwise logistic regression then was used to determine which potentially significant predictors provided the most explanatory power with regard to treatment response.

The sample size calculation was based on requirements of the multiple correspondence analyses using the formula N = Π_i = 1 à n _(M(i)) where M(i) is the number of modalities for variable i and n is the number of variables studied simultaneously. It was determined a priori that variables reflecting subject and disease characteristics, cardiovascular risk factors, and ophthalmic history would be entered into analyses. The target number of subjects to be enrolled was 768.

## Results

### Study population

**Figure 1:**
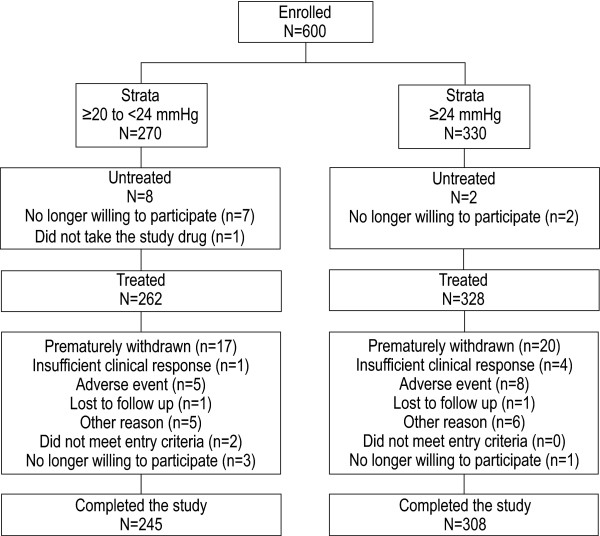
**Subject disposition**.

Between 22 December 2003 and 16 February 2005, 600 subjects with ocular hypertension or open-angle glaucoma were enrolled, 270 subjects in the 20 to <24 mmHg group and 330 subjects in the ≥ 24 mmHg group (Figure 1). In all, 590 subjects received ≥ 1 dose of latanoprost and were included in safety analyses (20 to <24 mmHg group, N = 262; ≥ 24 mmHg group, N = 328). The ITT population, which excluded 2 subjects who did not meet study entry criteria and 16 for whom no valid postbaseline IOP measurement was recorded, included 252 subjects in the 20 to <24 mmHg group and 320 subjects in the ≥ 24 mmHg group. In all, 553 subjects completed the study; the major reasons for premature withdrawal among the 590 treated subjects were adverse events and "other reasons" (n = 13/37 and n = 11/37, respectively; Figure 1).

Demographic and clinical characteristics of subjects at baseline are summarized in Table 1. The mean age of subjects in the two baseline IOP strata was similar, approximately 59 years; a larger percentage of those in the ≥ 24 mmHg group were male. More subjects in the ≥ 24 mmHg group were diagnosed with open-angle glaucoma, and, among those with glaucoma, a larger percentage in that group had disease classified as "severe."

**Table 1:** Baseline demographic and clinical characteristics, ITT population*

	Baseline IOP	
	
Characteristic	20 to <24 mmHg N = 252	≥ 24 mmHg N = 320
Age, years		
Mean ± SD	59.9 ± 12.2	59.1 ± 11.8
Gender, male	117 (46.4)	174 (54.4)
Diagnosis		
Ocular hypertension	150 (59.5)	161 (50.3)
Open-angle glaucoma	102 (40.5)	159 (49.7)
Open-angle glaucoma grade^†^		
Mild	77 (75.5)	96 (60.4)
Moderate	21 (20.6)	43 (27.0)
Severe	4 (3.9)	20 (12.6)
Intraocular pressure		
Mean (SD)	22.2 ± 0.9	26.7 ± 2.8
Family history of ocular		
hypertension or glaucoma	85 (33.7)	73 (22.8)
Body mass index		
Mean (SD)	25.0 ± 3.9	25.1 ± 3.9
Comorbidities^‡^		
Diabetes	17 (8.7)	33 (13.3)
Hypertension	69 (35.2)	77 (30.8)
Sleep disorders	39 (19.8)	34 (13.7)
Myopia	78 (31.0)	95 (29.7)
<-12 diopters	1 (1.3)	3 (3.2)
Between -6 and -12 diopters	9 (11.5)	13 (13.7)
>-6 diopters	68 (87.2)	79 (83.2)
Tobacco use within prior year^§^	55 (28.1)	83 (33.3)

*n (%) unless otherwise noted.

^†^Percentages based on number of subjects with open-angle glaucoma.

^‡^Percentages based on number of subjects reporting presence/absence of comorbidities.

^§^Percentages based on numbers of subjects reporting use/no use of tobacco within prior year.

ITT = intent to treat; SD = standard deviation.

Exposure to latanoprost was similar between groups: 93.1 ± 22.0 days in the 20 to <24 mmHg group and 91.6 ± 18.3 days in the ≥ 24 mmHg group. The majority of subjects reported high levels of compliance with once-daily latanoprost, with 98% in each group reporting that they took the medication either every day or that they "rarely" forgot.

### Efficacy

At month 3, mean IOP levels had decreased from baseline by -6.3 ± 2.4 mmHg in the 20 to <24 mmHg group and by -9.2 ± 3.7 mmHg in the ≥ 24 mmHg group (p < 0.0001 for each within-group comparison; Table 2). Mean IOP levels decreased rapidly from baseline to month 1 and remained stable through month 3 with percent IOP changes of -27.8 ± 10.3% and -28.0 ± 10.6%, respectively, in the 20 to <24 mmHg group and -32.9 ± 10.6% and -34.1 ± 11.9%, respectively, in the ≥ 24 mmHg group.

**Table 2:** Intraocular pressure (IOP) at baseline and month 3 (mmHg), ITT population

	Baseline IOP	
	
	20 to <24 mmHg N = 252	≥ 24 mmHg N = 320
Baseline IOP, mmHg		
Mean ± SD	22.2 ± 0.9	26.7 ± 2.8
Month 3 IOP, mmHg		
Mean ± SD	16.0 ± 2.3	17.5 ± 3.2
IOP change from baseline to month 3, mmHg		
Mean ± SD	-6.3 ± 2.4	-9.2 ± 3.7
p-value*	<0.0001	<0.0001
% change in IOP from baseline to month 3		
Mean ± SD	-28.0 ± 10.6	-34.1 ± 11.9

≥ 10% IOP reduction from baseline to month 3		
n (%)^†^	237 (94.1)	310 (96.9)

≥ 30% IOP reduction from baseline to month 3		
n (%)^‡^	122 (48.4)	210 (65.6)

*p-value based on *t *test for paired data.

^†^p = 0.10 for between-group difference; chi-square test.

^‡^p < 0.0001 for between-group difference; chi-square test.

ITT = intent to treat; SD = standard deviation.

**Figure 2:**
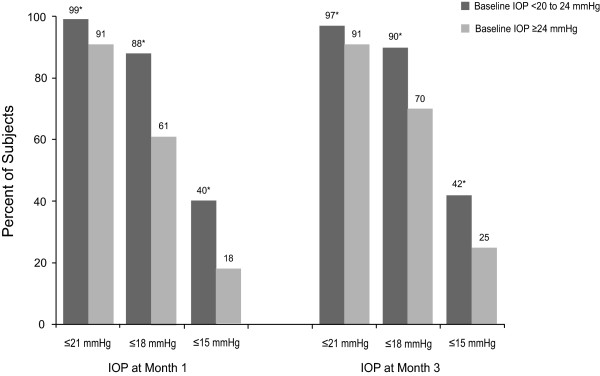
Subjects achieving prespecified mean intraocular pressure (IOP) levels at months 1 and 3 *p ≤ 0.001 for between-group difference.

Similarly large percentages of subjects achieved ≥ 10% IOP reductions from baseline to month 3 in both groups: 94.1% in the 20 to <24 mmHg group and 96.9% in the ≥ 24 mmHg group (p = 0.10; Table 2). A significantly smaller percentage of those in the 20 to <24 mmHg group achieved IOP reductions of ≥ 30% by month 3 (48.4% *vs *65.6%, respectively; p < 0.0001). In contrast, significantly smaller percentages of those in the higher IOP group achieved IOP levels ≤ 21, ≤ 18, or ≤ 15 mmHg at either months 1 or 3 (p < 0.001 for each between-group comparison; Figure 2). It is notable that 70% of subjects in the ≥ 24 mmHg group achieved IOP levels of ≤ 18 mmHg by month 3.

### Predictors of treatment response

Initial multiple correspondence analyses of the full variable set identified six potential predictors of response to treatment (IOP reduction ≥ 30% between baseline and month 3): age, gender, initial IOP stratum, current diagnosis, family history of ocular hypertension or glaucoma, and myopia. The logistic regression model with 568 subjects retained only two of these variables: initial IOP stratum and myopia. Treatment response was less likely in subjects whose baseline IOP was 20 to <24 mmHg (adjusted risk ratio [95% confidence interval [CI], 0.488 [0.346, 0.686]; p < 0.001) and less likely in those with myopia (≤-6 diopters *vs *none: adjusted risk ratio [95% CI]: 0.576 [0.391, 0.894]; p = 0.0053 and >-6 diopters *vs *none: adjusted risk ratio [95% CI]: 0.392 [0.175, 0.879]; p = 0.0230).

### Safety

Among the 590 subjects in the safety population, 237 (40.2%) reported ≥ 1 adverse events. Adverse event profiles were similar in subjects in the two baseline IOP strata (Table 3). Note that 119/339 (35.1%) adverse events and 119/235 (50.6%) treatment-related adverse events reflected subject report of latanoprost instillation from a bottle that had been opened for >28 days, which was documented as an adverse event; no ocular or systemic adverse event associated with use of latanoprost from an expired bottle was reported. In all, nine subjects reported 10 serious adverse events, none of which was considered related to study drug. No deaths were reported during the study.

**Table 3:** Summary of adverse events, safety population (N = 590)*

	Baseline Intraocular Pressure	
	
**Subjects with**:	20 to <24 mmHg N = 262	≥ 24 mmHg N = 328
≥ 1 adverse event^†^	111 (42.4) [159]	126 (38.4) [180]
≥ 1 treatment-related adverse event^†^	83 (31.7) [109]	99 (30.2) [126]
≥ 1 serious adverse event	5 (1.9) [5]	4 (1.2) [5]
≥ 1 adverse event leading to treatment discontinuation	5 (1.9) [7]	8 (2.4) [13]

*n (%) of subjects [number of events].

^†^Patient-reported use of a bottle of latanoprost beyond its expiration date, i.e., for >28 days after opening, was reported by 54 subjects in the 20 to <24 mmHg group and by 65 subjects in the ≥ 24 mmHg group. These were counted as adverse events and as treatment-related adverse events. No other ocular or systemic adverse event was associated with extended use in any subject.

The most frequent treatment-related ocular adverse event was conjuctival hyperemia, occurring in 10.7% (28/262) and 8.5% (28/328) subjects in the 20 to <24 and ≥ 24 mmHg groups, respectively (Table 4). Eye pain occurred in approximately 3% of subjects in each IOP stratum, and all other treatment-related ocular adverse events occurred in <2% of subjects.

**Table 4:** Number (%) of treatment-related ocular adverse events (N = 590)

	Baseline Intraocular Pressure	
	
**Subjects with**:	20 to <24 mmHg N = 262	≥ 24 mmHg N = 328
Hyperemia	28 (10.7)	28 (8.5)
Eye pain	8 (3.1)	11 (3.4)
Blepharitis	3 (1.2)	1 (0.3)
Eyelid pruritis	3 (1.2)	1 (0.3)
Eye abnormality NOS	2 (0.8)	2 (0.6)
Keratitis	2 (0.8)	2 (0.6)
Eyelid edema	0	3 (0.9)
Photophobia	1 (0.4)	0
Vision abnormal NOS	0	1 (0.3)
Xerophthalmia	0	1 (0.3)

NOS = not otherwise specified.

## Discussion

Short-term, prospective multicenter, randomized, double-masked trials have shown latanoprost instilled once daily to be at least as effective as and generally superior to timolol administered twice daily [9-13] and to be as effective and as safe as other frequently prescribed prostaglandin analogues [15-17]. In an observational study [18] of patients switched to latanoprost from previous glaucoma therapies, mean IOP was reduced from 21.3 ± 4.1 to 17.6 ± 3.2 mmHg in 1376 individuals followed for 2 years; the most common ocular adverse event was ocular irritation, noted in 1.6% of patients, while hyperemia was reported in 0.3%. A 5-year, open-label, safety surveillance study [19] included 5854 patients on IOP-reducing therapy other than latanoprost who required a change in therapy and who were randomly assigned to latanoprost or usual care (any other commercially available ocular hypotensive therapy). In both groups, serious adverse drug reactions were reported in approximately 0.4% of patients, and new occurrences of corneal erosions, iritis/uveitis, and macular edema were rare (risk ≤ 3.2% for each).

The present prospective, observational study extends these findings to "real world" treatment -naïve patients. We found first-line treatment with latanoprost to be effective and safe in ocular hypertension and open-angle glaucoma patients with pretreatment IOP levels in both the 20 to <24 mmHg range and ≥ 24 mmHg. Treatment response was rapid, seen within 1 month of initiating therapy, and was maintained throughout the follow-up period.

As would be expected, absolute and percentage IOP reductions were greater in the higher IOP stratum. Others [13,20-22] have observed that higher baseline IOP levels are associated with greater IOP reductions, perhaps in part, due to regression to the mean, the tendency for an extreme value at first measurement to be closer to the center of the distribution at later measurement time points [23]. Nevertheless, significant responses to latanoprost therapy were seen in both IOP strata. For example, IOP levels ≤ 18 mmHg were achieved by 90% and 70% of patients in the 20 to <24 and ≥ 24 mmHg groups, respectively, at month 3, and treatment response (≥ 30% IOP reduction from baseline to month 3) was observed in nearly one half and in two-thirds of patients, respectively. In this population of treatment-naïve patients, the percentage of responders in both groups was greater than the 30% which generally could be expected with prostaglandin monotherapy [24]. Moreover, more than 94% of patients in each stratum achieved ≥ 10% IOP reductions from baseline to month 3, a result consistent with that of a pooled-analysis of eight studies that found IOP reductions of >15% in 93% of glaucoma and ocular hypertension patients without prior glaucoma treatment other than prostaglandins after 3 to 6 months of latanoprost therapy [13].

In the multivariate model, IOP stratum and myopia were found to significantly predict treatment response while other candidate variables including age, gender, diagnosis, and family history did not. The predictive value of IOP stratum reflects the positive relationship between baseline IOP level and IOP reduction discussed above. It has been suggested that IOP reductions associated with latanoprost use could be greater in myopic eyes since a larger area of trabecular meshwork might be found in eyes with negative spherical equivalence [13]. We cannot explain our opposite finding that myopia was negatively associated with a treatment response. It is possible, however, that the relatively low proportion of patients in the higher myopia categories affected the results. Multivariate analyses in populations with a more even distribution of patients with myopia are needed.

As reported by previous researchers [9-13,15], latanoprost was safe and well tolerated with no important differences between IOP strata. Conjunctival hyperemia was the most frequently reported ocular adverse event, occurring in 9% of patients overall. In general, hyperemia is the most common adverse event associated with prostaglandin use [25-27]. More than one third of adverse events overall and one half of treatment-related adverse events reflected patient reports of instillation of latanoprost from bottles that had been opened for >28 days.

Previous research has demonstrated that substantial reductions in IOP levels [6-8,28] and, in particular, reductions to levels of <18 mmHg [5] delay or stop glaucomatous progression. For example, the Collaborative Initial Glaucoma Treatment Study [8] found that aggressive medical treatment resulting in diurnal IOP reductions of ≥ 35% from baseline virtually halted glaucomatous progression over 5 years, and treated patients in the Early Manifest Glaucoma Trial [7] had half the progression risk of untreated patients. Based on such findings, clinicians are encouraged when making treatment decisions to consider the likelihood of achieving a predetermined target IOP level or percentage IOP reduction consistent with the patient's disease status [24,29].

The relative convenience and side effect profiles of therapies also may be important factors in treatment decision making. More complex medication dosing schedules have been shown to negatively impact adherence in glaucoma patients [3,30-32]. Patients in the present study reported very high levels of treatment compliance with latanoprost. Although these compliance rates may to some extent reflect 'white-coat adherence' [33,34], patients prescribed once-daily prostaglandin analogues have been found to be more compliant [35] and to remain on treatment significantly longer [36-38] than those prescribed other classes of medication, including beta-blockers. Moreover, patients prescribed latanoprost are more persistent with therapy than those prescribed either bimatoprost or travoprost [37]. Ocular adverse events, especially hyperemia which is less common with latanoprost than with the other prostaglandin analogues, have been shown to negatively impact patient continuation with therapy [39].

The present study has a number of important strengths. First, findings of this prospective, open-label, uncontrolled, observational study better reflect actual clinical practice conditions than those based on randomized, controlled trials and therefore may be more generalizable to routine ophthalmology practices. Although the target of enrolling 768 subjects was not reached, the fact that 600 treatment-naive subjects were enrolled makes this, to our knowledge, the largest study of the effectiveness and safety of first-line latanoprost conducted to date. An additional strength is the study's inclusion of a multivariate model to evaluate potential predictors of treatment response.

The study's limitations include the fact that some investigators measured IOP levels using air-pulse tonometry while others used Goldmann applanation tonometry; ideally, all would have used the latter method. The relative infrequency of presentation by treatment-naive ocular hypertension or open-angle glaucoma patients to clinical practices necessitated the inclusion of a large number of centers to ensure timely subject recruitment; the impact on findings of interpractice variation in recording information and/or in treatment standards and practices is not known. Reflecting the study's observational, "real world" approach, investigators did not routinely record all variables of potential interest, such as central corneal thickness. In addition, the method used to measure IOP levels was not always documented making it infeasible to analyze IOP changes stratified by tonometry type. Finally, future research should include a larger population of treatment-naïve patients followed over a longer time period in order to ensure detection of rare but potentially serious adverse events; it is notable, however, that a large 5-year safety study [19] found rates of new cases of corneal erosions, iritis/uveitis, or macular edema to be low and similar in those treated with latanoprost or other approved ocular hypotensive therapies. A study with a larger patient population also would support an analysis of risk factors for nonresponse to latanoprost.

## Conclusions

This 3-month, open-label, multicenter, uncontrolled, phase IV, "real world" study found monotherapy with once-daily latanoprost to be effective and safe in treatment-naive ocular hypertension or open-angle glaucoma patients. Patients with baseline IOP levels of 20 to <24 mmHg as well as ≥ 24 mmHg benefitted from initial latanoprost treatment.

## Competing interests

Dr. Amrane was an employee of Pfizer Inc at the time the study was conducted. The remaining authors have no proprietary interests.

## Authors' contributions

PD participated in the study design, analysis and interpretation of data, drafting of the manuscript, critical revision of the manuscript for important intellectual content, and study supervision. CB participated in the study design, analysis and interpretation of data, and critical revision of the manuscript for important intellectual content. AB, JPN, and JPR participated in the study design, acquisition of data, and critical revision of the manuscript for important intellectual content. JFR and ES participated in the study design and critical revision of the manuscript for important intellectual content. MA participated in the analysis and interpretation of data, critical revision of the manuscript for important intellectual content, and study supervision. All authors read and approved the final manuscript.

## Pre-publication history

The pre-publication history for this paper can be accessed here:

http://www.biomedcentral.com/1471-2415/10/4/prepub
